# Enhancement of oral bioavailability of doxorubicin through surface modified biodegradable polymeric nanoparticles

**DOI:** 10.1186/s13065-018-0434-1

**Published:** 2018-05-23

**Authors:** Niyaz Ahmad, Rizwan Ahmad, Md Aftab Alam, Farhan Jalees Ahmad

**Affiliations:** 1 Department of Pharmaceutics, College of Clinical Pharmacy, Imam Abdulrahman Bin Faisal University, P.O. Box 1982, Dammam, 31441 Kingdom of Saudi Arabia; 2 Department of Natural Products and Alternative Medicine, College of Clinical Pharmacy, Imam Abdulrahman Bin Faisal University, Dammam, Kingdom of Saudi Arabia; 3 Department of Pharmaceutics, School of Medical and Allied Sciences, Galgotias University, Gautam Budh Nagar, Greater Noida, 201310 India; 4 Nanomedicine Lab, Department of Pharmaceutics, Faculty of Pharmacy, Jamia Hamdard, Hamdard Nagar, New Delhi, 110062 India

**Keywords:** Doxorubicin, PEG-PLGA-NPs, Oral drug delivery, Oral bioavailability, Plasma pharmacokinetic

## Abstract

**Background:**

Doxorubicin hydrochloride (DOX·HCl), an anthracycline glycoside antibiotic, exhibits low oral bioavailability due to active efflux from intestinal P-glycoprotein receptors. The oral administration of DOX remains a challenge hence; no oral formulation for DOX is marketed, till date.

**Aim of the study:**

To improve the oral bioavailability of DOX through, preparation of a nanoformulation i.e. PEGylated-doxorubicin(DOX)-loaded-poly-lactic-co-glycolic acid (PLGA)-Nanoparticles (NPs) and to develop and validate an ultra-high performance liquid chromatography electrospray ionization-synapt mass spectrometric bioanalytical method (UHPLC/ESI-QTOF–MS/MS) for plasma (Wistar rats) DOX quantification.

**Materials and methods:**

For chromatography, Waters ACQUITY UPLC™ along with a BEH C-18 column (2.1 mm × 100 mm; 1.7 μm), mobile phase conditions (acetonitrile: 0.1% formic acid::1:1 v/v) and flow rate (0.20 ml/min) was used. For analyte recovery from rat plasma, a liquid–liquid extraction method (LLE), using Acetonitrile: 5 mM ammonium acetate in a ratio of 6:4 v/v at pH 3.5, was used.

**Results:**

Nanoformulation with a particle size (183.10 ± 7.41 nm), zeta potential (− 13.10 ± 1.04 mV), drug content (42.69 ± 1.97 µg/mg) and a spherical shape and smooth surface was developed. An elution time of 1.61 and 1.75 min along with a transition at m/z 544.42/397.27 and 528.46/321.41 were observed for DOX and internal standard (IS) Daunorubicin, respectively. In addition, a linear dynamic range with r^2^ ≥ 0.9985 over a concentration range of 1.00–2500.0 ng/ml was observed for different processes and parameters used in the study. Similarly a marked improvement i.e. 6.8 fold was observed, in PEGylated-DOX-PLGA-NPs as compared to DOX-S, in pharmacokinetics studies.

**Conclusion:**

The promising approach of PEGylated-DOX-PLGA-NPs may provide an alternate to intravenous therapy for better patient care.

## Introduction

Doxorubicin hydrochloride (DOX·HCl), have been reported with a widespread applications in ovarian, breast and lung cancer as well as malignant lymphoma [1, 2] however, the cardiotoxicity associated side effect limits its long term use for such clinical purposes [3, 4]. The additional P-glycoprotein (P-gp) as well as multidrug-resistance-associated protein-1 (MRP1) mediated efflux, makes the tumor cells less sensitive towards DOX [5]. The disadvantages such as cardiac toxicity and short half-life [6, 7], poor solubility, lack of availability of oral dosage form (most invasive, cost effective and painless route), instability of drugs in gastric conditions and hepatic first pass effects hinder the use of most drugs [8].

To put an end for these gaps and improve the oral efficacy of drugs, various approaches in the form of polymer prodrugs [9, 10], polymer conjugates [11, 12], liposomes [13, 14], solid lipid NPs [15, 16] and polymeric nanoparticles (NPs) [17–19], have been evaluated. The successful outcomes for such approaches have been observed in the shape of SLN for doxorubicin [20, 21], layersomes for doxorubicin [22], SEDDS for etoposide [23], polymeric micelles for paclitaxel [24], dendrimer for doxorubicin [25] as well as polymeric NPs for etoposide and epirubicin respectively [26, 27]. PLGA-NPs was also investigated whereby an improvement in gemcitabine pharmacokinetic profile [28] as well as an enhanced pharmacodynamics profile was observed for doxorubicin and paclitaxel [29, 30]. In addition, PEG-decorated NPs have been reported to have a high diffusion property and penetration across thick layer of mucosa [21, 31] and an additive bio adhesive property [32, 33] which imparts the property of enhanced oral bioavailability as compared to non-PEGylated particles [21, 34]. Regarding PLGA-NPs, another study by Ahmad et al. [35], reported an enhanced bioavailability (~ 3–5.11 fold increase) for docetaxel across caco-2 cell line in rat ileum. In the study, PEGylated–PLGA-NPs for DOX (DOX-PEG-PLGA-PNPs), available in injectable form only for commercial purposes, will be developed and the surface-decoration-effects upon the PK and PD behavior of developed NPs will be evaluated. In addition to nanoparticle approaches, lack of improper less selective and sensitive method of quantification makes it difficult to measure DOX concentration in any biological samples, following oral administration. Though few attempts with conventional methods such as LC-fluorescence methods [36–39] and LC-UV method [40] have been reported, however none of the quantification method was successful to determine DOX concentration. A liquid chromatography/electrospray tandem mass spectrometry for quantification of PEG-liposomal-DOX was also developed by Arnold et al. [41], however lack of selectivity was observed for the method. Literature for DOX-plasma-quantification is though available [42–46], but they suffer major drawbacks such as; no individual or separate method for DOX plasma determination, lack of sensitivity and ability to determination at picogram level.

Hereby, the study aims to develop and validate a rapid, selective, sensitive and robust method using UPLC-ESI-Q-TOF-MS/MS for quantification of DOX-PEG-PLGA-PNPs in rat plasma. The method developed is carried out with a particular emphasis to minimize the carry over effect and to determine the plasma-DOX concentration (picogram level) in developed NPs. A nano formulation with enhanced DOX-oral bioavailability in plasma at low doses and a bioanalytical method for determining its picogram level is the main focus for the study.

## Materials and methods

### Materials

Daunorubicin hydrochloride and doxorubicin hydrochloride were provided by Jubilant Chemsys Ltd. Noida, Uttar Pradesh, India (purity ≥ 98%). PLGA was purchased from Supreme Combine, Mumbai, India and 1-(3-dimethylaminopropyl)-3-ethylcarbodiimide hydrochloride (EDC) was purchased from Thermo Scientific. Dichloromethane (DCM) was obtained from Qualigens Fine Chemical, Mumbai, India. Polyvinyl alcohol (PVA, MW-25,000; 16,000), and sodium tripolyphosphate (TPP), ammonium formate (MS grade), acetonitrile and methanol (LC–MS grade), ammonium acetate (MS grade) and formic acid (purity > 98%) were obtained from Sigma-Aldrich (St Louis, MO). Water used was purified through Milli-Q water purification system (Millipore, Bedfrod, MA).

### Preparation of doxorubicin surface-modified PLGA-polymeric nanoparticles

Single emulsion (o/w)–solvent evaporation technique, adopted from Ahmad et al. [35], was applied for preparation of DOX-NPs. Briefly; drug was dissolved in a dichloromethane (DCM) dissolved PLGA in order to obtain a final concentration of 10 mg/100 ml (drug/PLGA solution), added to an aqueous phase (1% w/v PVA) with proper sonication (1 min, 30% voltage efficiency, 25 °C) and the emulsion thus produced was subjected to a mechanical stirring (15 min at 6000 rpm) and evaporation of DCM under vacuum (Hahn Shin Science Co., Gyeonggido, South Korea). Using a centrifugation process for 30 min at 15,000 rpm (REMI, Mumbai, India) and cold distilled water washing, PEGylated-DOX-loaded-PLGA-NPs were made apart from bulk aqueous phase and freeze dried (Labconco, TriadTM, Kansas City, MO). In addition, PEGylation was produced for developed PLGA-NPs using an EDC coupling reaction technique as reported [47].

#### Nanoparticles size, size distribution, zeta potential

Dynamic light scattering technique (DLS) coupled with a computerized inspection system (Malvern Zetasizer, Nano-ZS, Malvern, UK) and ‘DTS nano software’ was used to determine size, polydispersity index (PDI) and zeta potential of develop NPs.

#### Shape and surface morphological analysis

Surface morphology for developed NPs, using TEM technique (Morgagni 268D; FEI Company, Hillsboro, OR), was determined as; putting a drop of nanosuspension for 1 min/60 s (in order to stick) over a paraffin sheet covered with copper grid, thereafter placing the grid in a phosphotungstate drop (> 5 s) and samples air dried and subjected again to TEM.

For surface texture determination of optimized PEGylated-DOX-loaded-PLGA-NPs, using SEM technique (Zeiss EVO40; Carl Zeiss, Cambridge, UK), sample was make spread over a conductive tape (double sided) and stucked with surface using SCD020 Blazers sputter coater unit (BAL-TEC GmbH, Witten, Germany) under high vacuum with gold whereas the environment of the coater was pre-maintained with the help of Argon gas (50 mA; 100 s).

#### Entrapment efficiency (EE) and drug loading capacity (LC)

PEGylated-DOX-loaded-PLGA-NPs were subjected to an ultracentrifugation technique (15,000 rpm at 4 °C for 30 min) for estimation of LC and EE whereas for free DOX-plasma analysis, a developed and validated UPLC-MS/MS method was utilized. The chromatographic conditions used were as; mobile phase i.e. acetonitrile and 0.01% formic acid (50%:50% v/v), and flow rate of 0.2 ml/min. Following a triplicate measurement, EE and LC were determined as [48];

$${\text{EE}}\% = \frac{{\left( {{\text{DOX}}_{\text{total}} - {\text{ DOX}}_{\text{free}} } \right)}}{{{\text{DOX}}_{\text{total}} }} \times 100$$

$${\text{DL}}\% = \frac{\text{Amount of entrapped DOX}}{\text{Total weight of nanoparticles}} \times 100$$

The yield for PEGylated-DOX-loaded-PLGA-NPs was calculated as;

$${\text{Process yield }}\left( \% \right) \, = {\text{W}}_{ 1} /{\text{W}}_{ 2} \times 100$$

W_1_ = recovered dried NPs weight, W_2_ = sum of initial dry weight of starting material.

#### In vitro release modeling

Dialysis bag method (Spectra/Por^®^ Spectrum Laboratories, Inc. Rancho Dominguez, CA, USA; MW cut off of 8–10 kDa), was applied to determine the in vitro release for DOX as; DOX (2 mg) and PEGylated-DOX-loaded-PLGA-NPs (equivalent to 2 mg of DOX) were dispersed in dissolution media (5 ml), added to dialysis bag and finally immersed in dissolution media (50 ml). An incubator shaker (SI6R, Shel Lab, Sheldon Mfg. Inc. Ave, Cornelius, OR, USA), controlled with proper stirring speed (100 rpm) and temperature (37 ± 0.5 °C), was used to study the in vitro release, whereas for dissolution studies a simulated gastric fluid (pH 1.2) and an intestinal fluid (pH 6.5) environment was used for 2 and 48 h thereafter, respectively. Samples were collected at properly scheduled time interval of 0.25, 0.5, 1, 2, 3, 4, 6, 8, 12, 24 and 48 h and subjected to an in-house developed UPLC-ESI-Q-TOF–MS/MS method, for further analysis.

### Bioanalytical method development and validation

#### UHPLC conditions

The optimized UHPLC conditions consisted of; a C-18 column (Waters ACQUITY UPLC™ BEH) with dimensions i.e. 2.1 mm × 100 mm; 1.7 µm, Acetonitrile: 0.1% Formic acid (50%/50%, v/v) as mobile phase with isocratic elution, flow rate (0.20 ml/min), injection volume (10 µl) and run time (4.0 min). The instrument used was Waters ACQUITY UPLC™ (Waters Corp., MA, USA) with attached binary solvent delivery system and tuneable MS detector.

#### ESI-Q-TOF–MS conditions

To perform MS, Waters Q-TOF Premier mass spectrometer system (Micromass MS Technologies, Manchester, UK) was utilized with operating conditions as; V-mode, resolution (above 32000 mass), scan time (1.0 min), collision gas (argon at a pressure of 5.3 × 10^−5^ Torr) and inter-scan delay (0.02 s). For quantification; Synapt mass spectrometry (Synapt MS) with trap collision energy i.e. Trap CE at 10.0 and 16.21 eV, showed a transition at m/z 544.42/397.27 for DOX and 528.46/321.41 for Daunorubicin (IS), as shown in Figs. 4 and 5 whereas for an accurate mass determination of precursor and fragment ion, Mass Lynx software (V 1.4) was used.

#### Quality control (QC) sample and standard sample preparation

A standard DOX-stock solution in methanol (10 mg/ml) was prepared and sonicated (20 min at 44 kHz/250 W). For calibration curve (CC); aqueous analyte (2%) was spiked in blank-rat-plasma (aqueous aliquots) i.e. 20 ml + 980 ml, respectively thus obtaining eight non-zero concentrations (A–H) for DOX i.e. 1–2500 ng/ml with an individual analyte concentrations of 1, 2, 40, 540, 1060, 1600, 2150 and 2500 ng/ml. For QC samples, four independent levels were prepared as; high quality control (HQC i.e. 2000 ng/ml), middle quality control (MQC i.e. 1000 ng/ml), low quality control (LQC i.e. 2.9 ng/ml) and lower limit of quality control (LLOQC i.e. 1.01 ng/ml). In addition, an internal standard (IS) solution was prepared through dilution of stock solution in methanol: water mixture i.e. 1:1. All solutions were stored at 2–8 °C, until used.

#### Sample preparation protocol

The solutions i.e. QC samples, CC standards and unknown plasma samples, were freshly prepared as; each sample (600 µl aliquot) alongwith a 50 µl IS (50 ng/ml) was taken in a glass tub, 5% formic acid (200 µl) solution was added (breaking protein binding) and vortexed (300 rpm, 5 min). A separately prepared extraction mixture (5 ml), consisting of Acetonitrile: 5 mM ammonium acetate (6:4 v/v, pH 3.5), was added to the mentioned prepared samples followed by shaking (20 min at 100 rpm) using a reciprocating shaker. A centrifugation process was used where the tubes were placed in centrifuge machine and allowed to spin (10 min at 4000 rpm and 4 °C). The supernatant thus obtained (4 ml), was preserved in another clean glass tube and dried with the help of Nitrogen stream (< 20 psi; temperature 50 ± 2.0 °C), dissolved in mobile phase (600 µl) and transferred to small vials (10 µl) for further analysis.

### Bioanalytical method validation

To validate the bioanalytical method for DOX in plasma, US-FDA guidelines were followed as reported [35, 49–51, 59], whereas three standard plots (containing eight non-zero concentrations) were analysed in order to determine method linearity. The calibration curve was constructed as; selecting analyte/IS peak area ratio via weighted linear least squares regression (1/x^2^) for plasma concentration alongwith measured peak area ratio. LLOQ i.e. lowest concentration of the calibration curve, measured through accuracy and precision, was determined from signal (10): noise (1) ratio. DOX recovery (extraction efficiency), measured through mean area from six replicates of extracted samples vs. extracted drug free plasma samples (both spiked before extraction), was determined at each individual levels (LQC, MQC and HQC). The same method was used to estimate IS recovery. In order to investigate intra-day accuracy and precision; DOX-plasma-samples replicate analysis was performed whereby six replicate samples from LLOQC, LQC, MQC, and HQC as well as their calibration curve were selected in the run. For analysis of inter-day precision and accuracy; six separate batches on three consecutive d were analyzed. For robustness of the method, changes in operating conditions such as composition, pH and flow rate of mobile phase for selected LQC, MQC and HQC levels of QC samples whereas for ruggedness of the method, different factors such as use of different columns (within same manufacturer), running the sample on same instrument while changing the analyst, were evaluated on one batch of precision and accuracy. Six replicates for LLOQC, LQC, MQC, and HQC samples were used in this run.

#### Matrix effect

To observe the effect of matrix on analyte quantification, six samples from different plasma batches prepared at LQC and HQC level, were analyzed for % accuracy and precision (%CV). The value (back calculated) obtained from QC’s nominal concentration were considered for assessing matrix effect whereas Post-extraction-spiking method was applied to note the matrix effects, in properly stored samples.

Matrix effect was calculated through the ratio i.e. A/B × 100, where A = peak area of the analyte i.e. MQC (spiked-blank-plasma with a known concentration) and B = corresponding peak area i.e. produced following the injection of standard in the mobile phase.

#### LOD and LOQ

LOD and LOQ were calculated from the standard deviation (SD) responses obtained after injections of blank mobile phase in triplicate and the slope of calibration (S). To determine LOD and LOQ experimentally; known concentration of DOX were diluted till the average responses were almost three or ten times the SD response for triplicate. The formulae used for the calculations were as;

$$LOQ = \frac{Std.Deviation \times 10}{Slope}$$

$$LOD = \frac{Std.Deviation \times 3.3}{Slope}$$

#### Ex-vivo stability

For DOX ex vivo stability, six plasma replicates for LQC (concentrations = 2.9 ng/ml) and HQC (concentration = 2000 ng/ml) exposed to different sets of temperature and time were analysed. The % age stability was determined as;

$${\text{Stability }}\left( \% \right) = \frac{\text{Mean corrected response of stability stock}}{\text{Mean response of comparison stock}} \times { 1}00$$

#### Long-term stability

To assess long term stability, six plasma replicates of LQC and HQC stored at deep freezer (30 days, − 40 °C) and standard-spiked-plasma sample were used.

#### Freeze–thaw stability

For freeze thaw stability, the same six plasma replicates of LQC and HQC were used for evaluation, however, the samples were treated three times with consecutive freeze thaw cycles (from − 40 °C to room temperature ± 25 °C).

#### Bench-top stability

Six plasma samples of LQC and HQC were stored at optimized conditions (24 h) and thereafter used to determine bench-top stability whereas quantification of QC samples was done against freshly-spiked-calibration curve standards.

#### Post-processing stability

For short term stability, six-plasma sets (each of LQC and HQC samples) were considered for analysis; however, the samples were pre-exposed to a temperature of 10 °C in an autosampler (24 h). Samples were processed and analyzed after specified storage conditions and the analyte with a precision (below 15%) and accuracy (85–115%) were considered stable [35, 50].

### In vivo study

#### Experimental animal

For in vivo studies, a proper approval was sorted from Animal Ethical Committee, Jamia Hamdard (New Delhi, India) whereas the animals i.e. Wistar rats (n = 6, age: 8–10 weeks, weight: 250–400 g) were kept (12 h dark–light cycle) in an environment properly controlled with regard to room temperature (25 ± 2 °C) and humidity (60 ± 5%). Standard pellet diet as well as water was used for feeding the animals, however, the animals were kept on fasting before any experiment.

#### Pharmacokinetic (PK) study

Rats (2 × 6 = 12) were administered with DOX (10 mg/kg orally) however, first group received DOX-S whereas second group was treated with DOX-loaded-PEGylated-PLGA-NPs suspension. Blood samples (0.2 ml) at specified time intervals i.e. 0.5, 1, 2, 3, 4, 6, 8, 12, 24, and 48 h were withdrawn from retro orbital choroid plexus (under mild anesthesia), collected in EDTA-tubes and finally the plasma was separated through centrifugation (rpm = 400, time = 10 min) and stored (−  40 °C), until further analysis through UPLC-ESI-Q-TOF–MS/MS using the developed in-house method.

### Statistical analysis

All the data was expressed as mean and standard deviation (SD), whereas a one-way analysis of variance (ANOVA) test at P < 0.05, was applied to analyze and compare the date using GraphPad InStat software (v 3.00, GraphPad Software, San Diego, CA).

## Result and discussion

### Preparation of PEGylated-PLGA-nanoparticles and their characterization

A single-emulsion solvent evaporation technique, due to DOX high water solubility, was employed to develop DOX-PLGA-NPs whereas an internal acidified aqueous phase, due to basic character of DOX which is more soluble in acidic medium, with a pH 3 was selected in order to have an improved encapsulation. Furthermore, PVA (1% w/v) alongwith a pH adjustment for internal phase was undertaken in order to enhance viscosity and reduce leaching effect as observed mostly for hydrophilic drugs. As the addition of PEG to DOX-loaded-PLGA-NPs enhances water permeability via PEG coating [47] thus the particles hereby were decorated according to manufacturer’s instructions i.e. using methoxypolyethylene glycol amine through EDC coupling reaction. EDC contains a cross linking between carboxyl and amine-reactive agents which helps activate carboxyl group and reacts with an amino group to form amide bond hence they are mostly applied for NPS surface decoration.

**Fig. 1:**
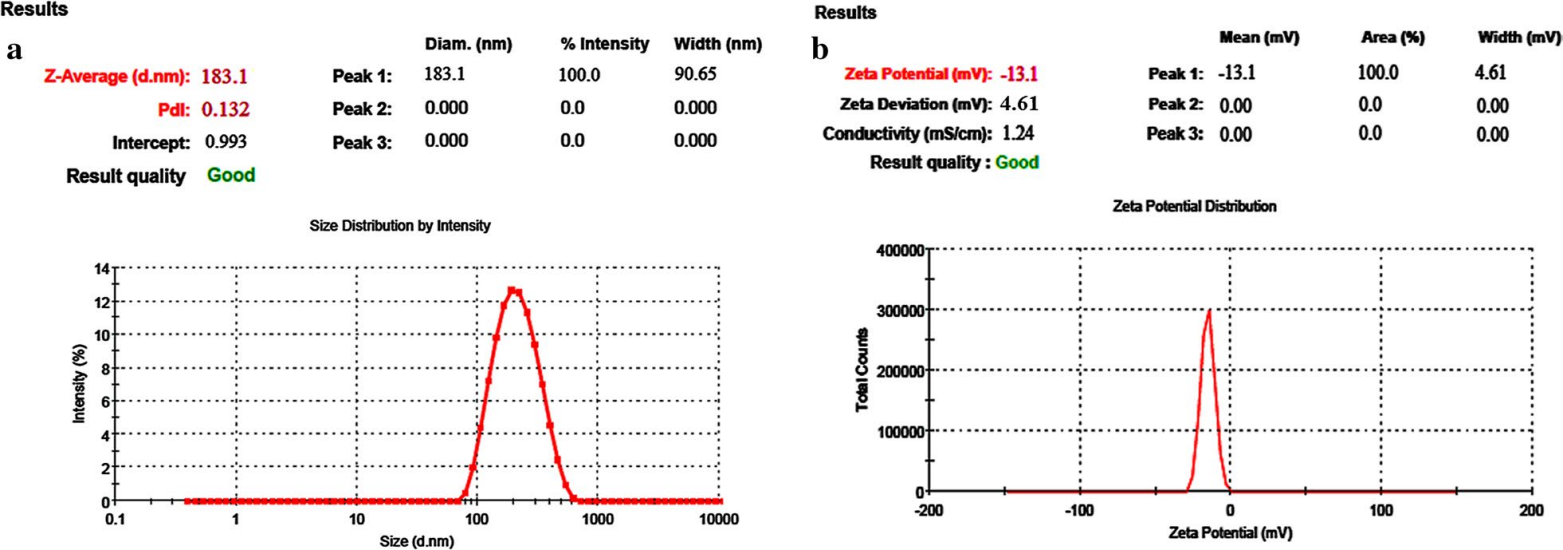
Dynamic light scattering techniques for determining the particle size distribution of DOX-loaded-PEG-PLGA-NPs Globule Size, (**a**) and zeta potential, (**b**)

**Fig. 2:**
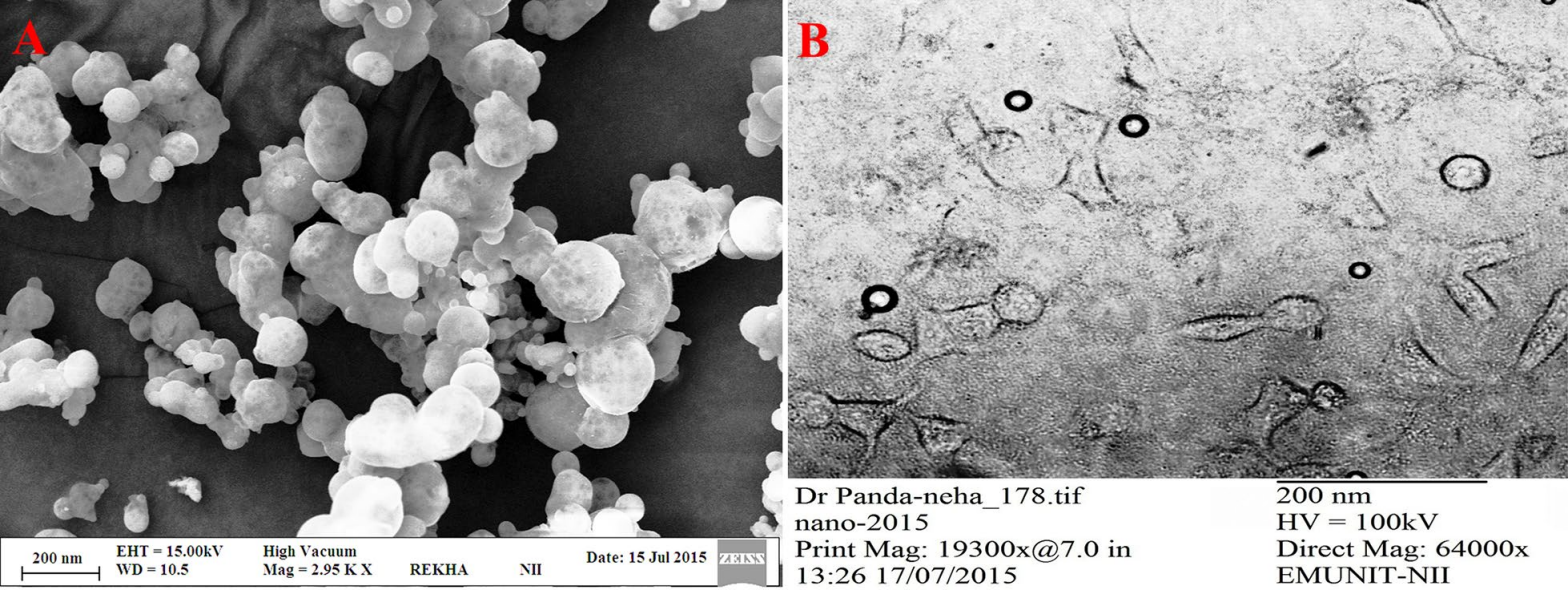
Scanning electron microscopy (SEM) (**A**) and Transmission electron microscopy (TEM) (**B**) images of DOX-loaded-PEG-PLGA-NPs

The developed PEGylated-DOX-PLGA-NPs were subjected to Malvern Zeta sizer for determination of particle size and size distribution whereby an average particle size (183.10 ± 7.41 nm) with a narrow PDI (0.132 ± 0.010) was observed, as shown in Fig. 1a. The zeta potential (− 13.10 ± 1.04) for developed PEGylated-DOX-PLGA-NPs as shown in Fig. 1b, was less negative as compared to simple PLGA-NPs, resulted due to free carboxylic group blockade on PLGA (amide bond formation between carboxylic and amine group of methoxypolyethylene glycol amine). Change in zeta potential [52] as well as FTIR, further confirmed the attachment for decorating molecules. Furthermore, a reasonable and considerable drug content i.e. 42.69 ± 1.97 µg/mg (process yield = 89.96 ± 3.05%, entrapment efficiency = 76.86 ± 3.51%, drug loading = 5.68 ± 0.09%) was observed for PEGylated-DOX-PLGA-NPs [35, 53]. Furthermore, SEM and TEM (Fig. 2A, B) showed a particle size (145–200 nm) in agreement with DSL results whereas a spherical shape with smooth surface for developed NPs was found through micrographs.

#### In vitro doxorubicin drug release study

**Fig. 3:**
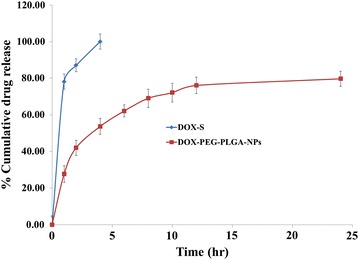
In vitro release profile of DOX-Solution and DOX-loaded-PEG-PLGA-NPs performed by using dialysis bag method, revealing sustained release pattern of DOX-loaded-PEG-PLGA-NPs (mean ± SD, n = 3)

A cumulative percentage release pattern was observed for DOX during in vitro study as; a total release of 98.09 ± 2.06% for DOX-S during the first 4.0 h, followed by a release of 79.56 ± 5.01% for PEG-DOX-PLGA-NPs within 24 h, as shown in Fig. 3. Basically, a biphasic release was observed for PEGylated-DOX-PLGA-NPs whereby an initial release of 36.71 ± 2.14% in first 2 h followed by a sustained release was seen for total drug. This initial burst may be suggested due to the properties of; being more soluble in dissolution media as well as availability of the drug near to NPs surface thus enhancing its quick diffusion whereas the presence of drug molecules at center and densely embedded polymer matrix as well as longer travel/diffusion path for the molecules present at NPs core, favored the sustained effect. Researchers view this initial burst release of drug an advantageous factor as it may help achieve an immediate therapeutic concentration while on the other hand an extended therapeutic effect is observed from sustained release pattern.

### Bioanalytical method development and validation by UPLC/ESI-Q-TOF–MS/MS

**Fig. 4:**
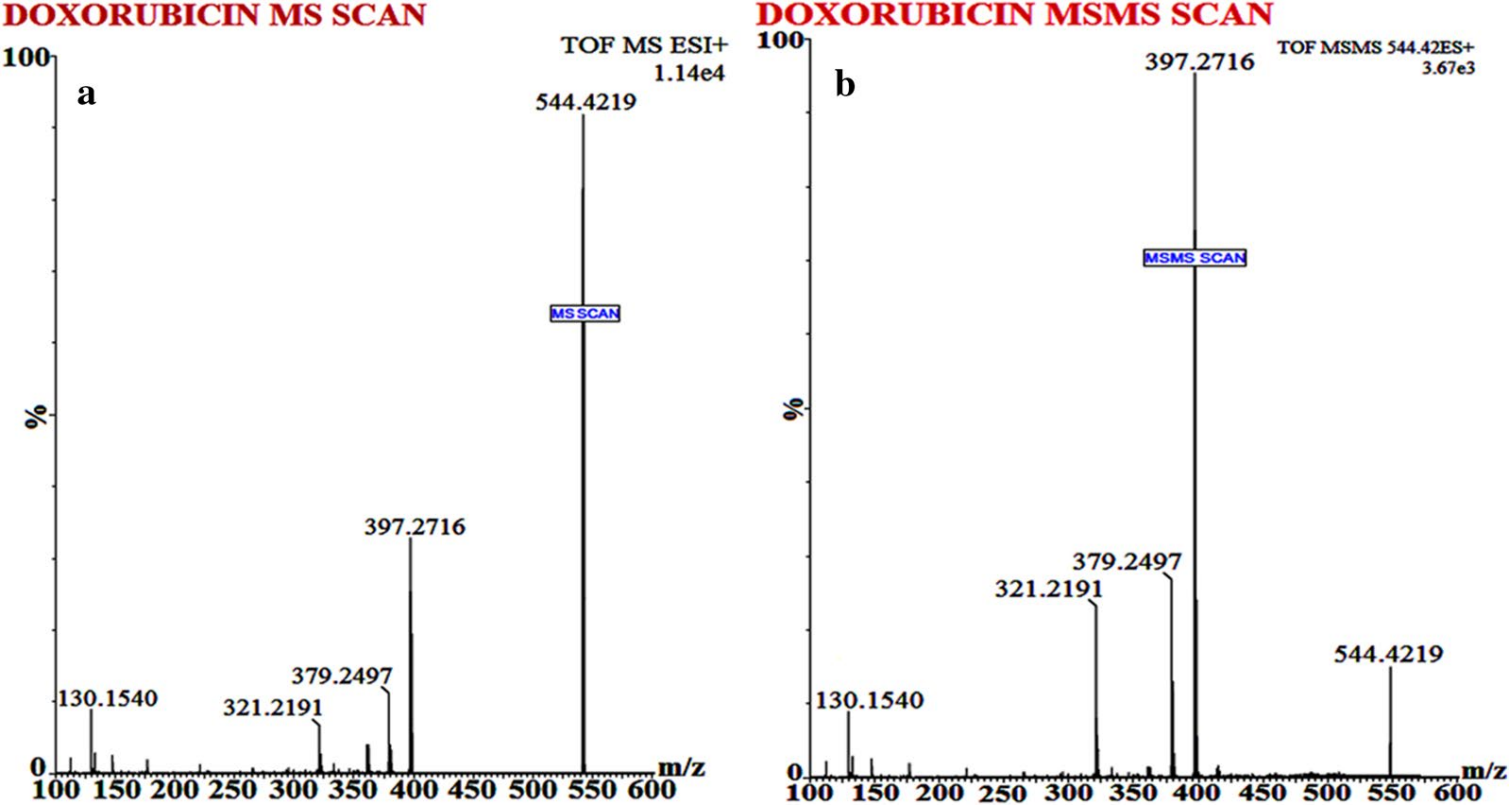
Mass spectrum of **a** doxorubicin parent ion (protonated precursor [M−H]^+^ ions at m/z 544.42) and **b** doxorubicin product ion (major fragmented product ion at m/z 397.27) showing fragmentation transitions

**Fig. 5:**
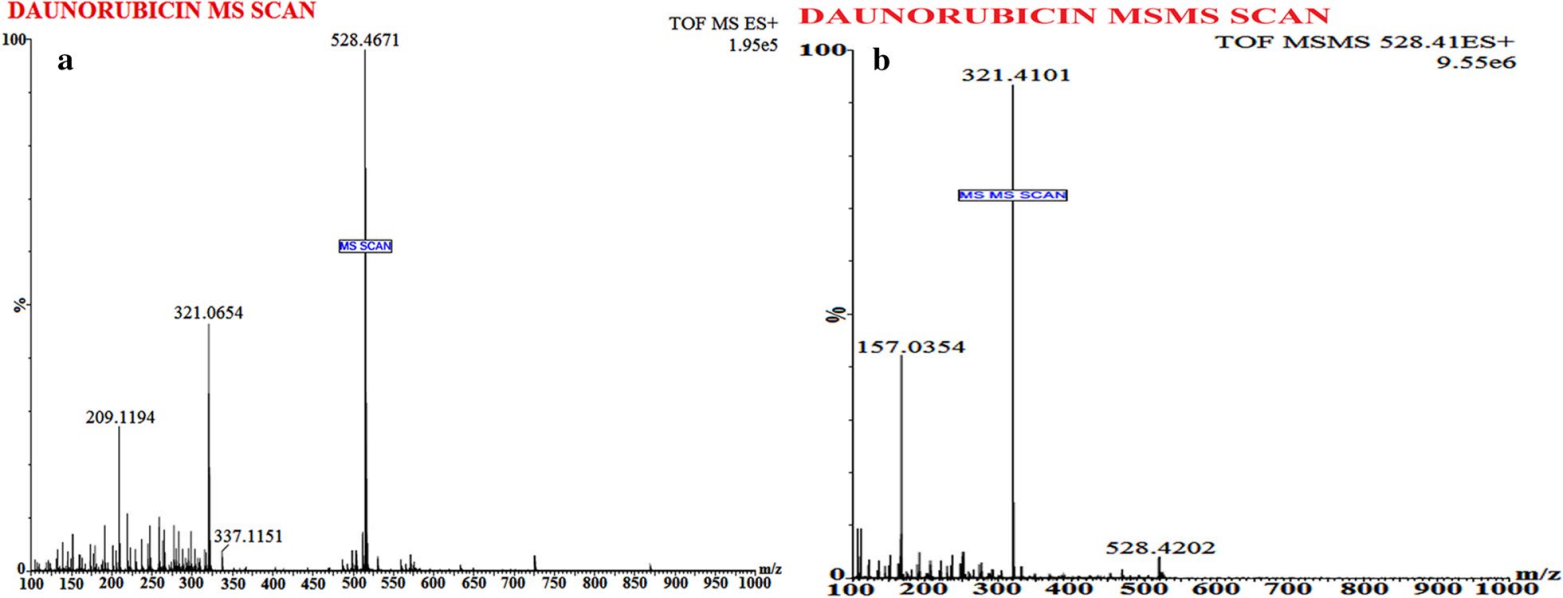
Mass spectrum of, **a** daunorubicin (IS) precursor ion (protonated precursor [M−H]^+^ ions at m/z 528.46 and **b** IS product ion (major fragmented product ions at m/z 321.41) showing fragmentation transitions

The low molecular weight and presence of alcoholic group in DOX structures offers the advantage to detect DOX easily in positive ion mode. A variety of solvents (methanol and isopropyl alcohol etc.) as well as buffer systems (ammonium formate (10–15% v/v), ammonium acetate (10–15% v/v)) was tried for bioanalytical method development but none of the mentioned components resulted in an efficient chromatographic resolution and sharp peak, respectively. Following an in-depth trial (change of solvents and buffers) an optimized chromatographic condition was developed as; mobile phase i.e. acetonitrile (50%):0.1% Formic acid (50%) v/v with a flow rate of 0.20 ml/min over a total run time of 4.0 min. The MS spectra showed protonated molecules at m/z 544.42/397.27 (Fig. 4a, b) and 528.46/321.41 (Fig. 5a, b) for DOX and IS, respectively whereas an optimum collision energy of 10.0 eV (DOX) and 16.21 eV (IS) as well as a capillary voltage (4.5 kV) was applied, in order to monitor precursor ions.

**Fig. 6:**
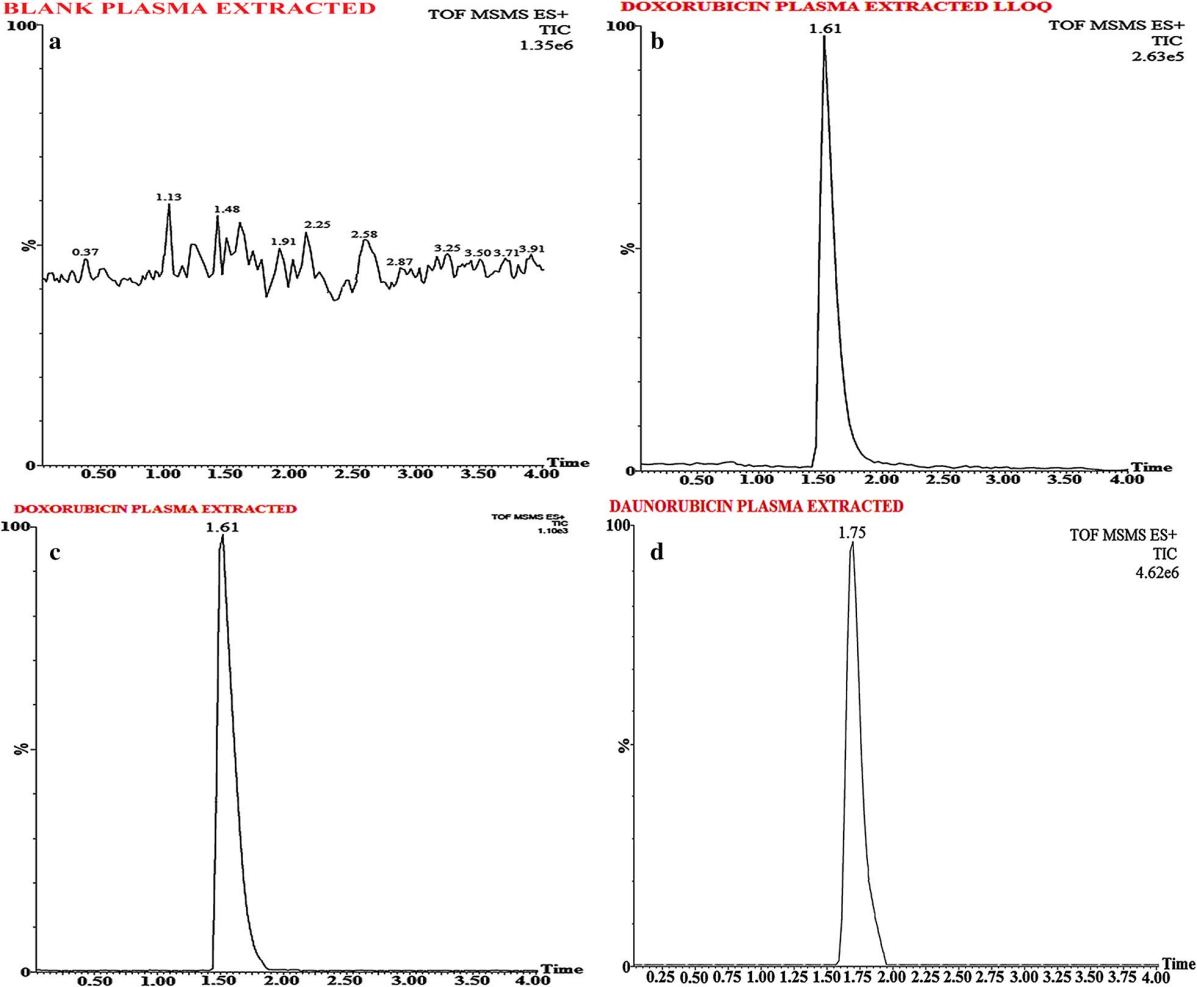
Typical chromatograms of extracted blank plasma (**a**), Plasma extracted doxorubicin (DOX) LLOQ (**b**), Plasma extracted doxorubicin (DOX) (**c**), and Plasma extracted daunorubicin (**d**) (IS)

**Fig. 7:**
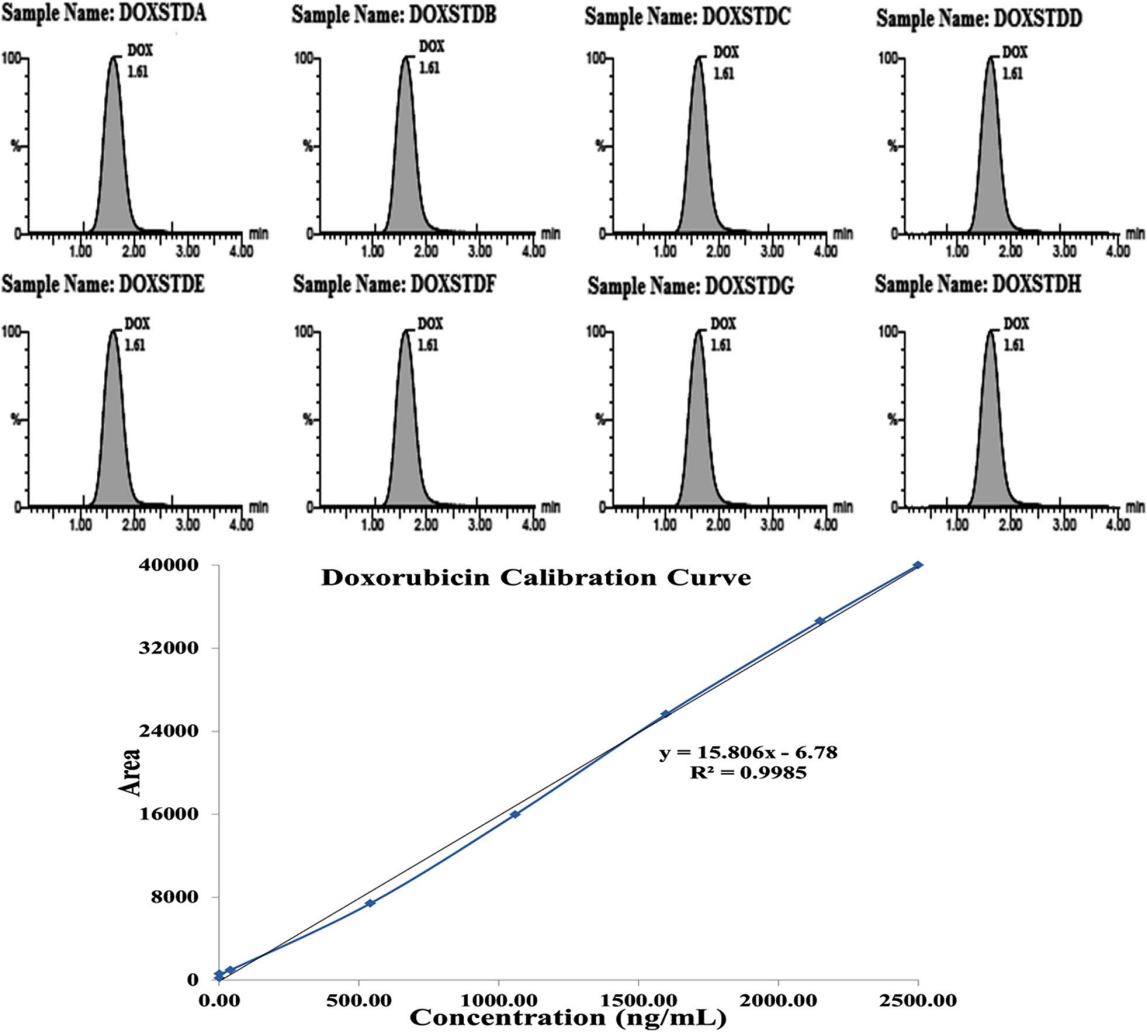
Calibration standard peaks at different calibration standards 1.0, 2.0, 40.0, 540.0, 1060.0, 1600, 2150, and 2500.0 ng/ml with their calibration graph (1.0–2500.0 ng/ml)

For biological sample preparation, different techniques such as liquid–liquid extraction (LLE), solid-phase extraction (SPE) and protein precipitation (PPT) are available however PPT, due to ion suppression of the endogenous substance in rat plasma, was not applied for API separation. Though ion suppression issue may resolve with chromatographic separation however it will increase run time. Following the trials with other extraction techniques, LLE was found the most efficient for DOX-striatum sample preparation. For maximum recovery, seven solvents (chloroform, acetonitrile, tertiary butyl methyl ether (TBME), Ethyl acetate, diethyl ether, n-hexane and dichloromethane) were applied for extraction however none of the solvent was able to achieve maximum recovery alone. On contrary, highest recovery (> 84.89%) was found for DOX and IS with the use of a mixture of solvents i.e. Acetonitrile (6%): 5 mM ammonium acetate (4%) v/v. A chromatogram for blank plasma i.e. extracted and reconstituted as shown in Fig. 6a, whereas elution time for DOX-plasma sample (1.61 min) (Fig. 7) and IS (1.75 min) is shown in Figs. 5d, 6b, c.

#### Linearity

The calibration curve for DOX showed; linearity (concentration range 1.00–2500.00 ng/ml), r^2^ ≥ 0.9985 (least squares regression) (Fig. 7) and accuracy and precision (%CV) as 94.06–98.94 and 1.10–2.33%, respectively (Table 1).

**Table 1:** Precision and accuracy data for doxorubicin (DOX)

Intra-batch					Inter-batch		
QC ID	Theoretical content (ng/ml)	Mean concentration observed (ng/ml)	Accuracy^a^ (%)	CV^b^ (%)	Mean concentration observed (ng/ml)	Accuracy^a^ (%)	CV^b^ (%)
LOQQC	1.01	0.98 ± 0.012	97.03	1.22	0.95 ± 0.020	94.06	2.11
LQC	2.90	2.85 ± 0.060	98.28	2.11	2.83 ± 0.066	97.59	2.33
MQC	1000.00	981.04 ± 15.04	98.10	1.53	977.37 ± 16.01	97.74	1.64
HQC	2000.00	1978.89 ± 23.93	98.94	1.21	1971.05 ± 21.67	98.55	1.10

Values (Mean ± SD) are derived from 6 replicates:

^a^ Accuracy (%) = Mean value of [(mean observed concentration)/(theoretical concentration)] × 100

^b^ Precision (%): coefficient of variance (percentage) = standard deviation divided by mean concentration found × 100; Theoretical contents; LOQQC: 1.01 ng/ml, LQC: 2.90 ng/ml; MQC: 1000 ng/ml; and HQC: 2000 ng/ml

#### Accuracy and precision

No interference with retention time of analyte and IS, from any endogenous peak, was noted from any batch. The representative chromatogram from both i.e. IS fortified plasma extract as well as DOX fortified blank plasma, showed a selectivity of the method as presented in Fig. 6a. A mean recovery for DOX (spiked plasma, n = 6) at different QC levels were observed as; 84.89% (LQC), 86.03% (MQC) and 85.54% (HQC) whereas a recovery of 84.96% was observed for IS. A precision (%CV) within a range of 1.21–2.11% and 1.10–2.33% for intra-batch and inter-batch as well as accuracy within the range of 97.03–98.94 and 94.06–98.55%, for intra-batch and inter-batch, was observed at QC levels for all DOX samples (Table 1).

#### Robustness

The effect of various parameters upon the CV (% age) and RT recovery, was evaluated for suitability of the system. These parameters consisted; change in mobile phase (Acetonitrile: 0.1% formic acid) concentrations i.e. 49.9:50.10, 50:50, 50.10:49.90 v/v, change in mobile phase pH i.e. 6.9, 7.2 and 7.4 and flow rate variability i.e. 0.19, 0.20 and 0.21 ml/min. The robustness for the method was achieved through small modifications in our developed method whereby a low value for DOX (0.42–4.44%) in terms of %CV was observed, as shown in Table 2a.

**Table 2:** Robustness of the method for doxorubicin (DOX)

(a) Robustness			
Conditions	LQC (2.90 ng/ml)	MQC(1000 ng/ml)	HQC (2000 ng/ml)
Mobile phase [ACN: 0.1% formic acid (50:50: v/v)]			
Negative level (49.9:50.10, n = 3)	2.70 ± 0.11 (4.07%)	970.10 ± 11.01 (1.13%)	1967.89 ± 15.09 (0.77%)
Zero level (50:50, n = 3)	2.84 ± 0.02 (0.70%)	981.89 ± 13.99 (1.42%)	1988.99 ± 11.89 (0.60%)
Positive level (50.10:49.90, n = 3)	2.75 ± 0.12 (4.36%)	974.91 ± 12.08 (1.24%)	1970.01 ± 14.09 (0.72%)
Flow rate (0.20 ml/min)			
Negative level (0.19, n = 3)	2.61 ± 0.09 (3.45%)	969.01 ± 11.33 (1.17%)	1971.61 ± 19.02 (0.96%)
Zero level (0.20, n = 3)	2.79 ± 0.081 (2.90%)	988.78 ± 12.01 (1.21%)	1981.01 ± 12.91 (0.65%)
Positive level (0.21, n = 3)	2.70 ± 0.12 (4.44%)	972.05 ± 10.56 (1.09%)	1967.29 ± 11.56 (0.59%)
pH of mobile phase (default pH = 7.2)			
Negative level (6.9, n = 3)	2.61 ± 0.061 (2.34%)	971.98 ± 5.99 (0.62%)	1968.45 ± 11.05 (0.56%)
Zero level (7.2, n = 3)	2.84 ± 0.012 (0.42%)	985.11 ± 8.92 (0.91%)	1985.42 ± 10.66 (0.54%)
Positive level (7.4, n = 3)	2.69 ± 0.058 (2.16%)	974.91 ± 6.30 (0.65%)	1977.00 ± 15.99 (0.81%)

(b) Ruggedness				
QC ID	Theoretical content (ng/ml)	Mean concentration observed (ng/ml)	Accuracy ^a^ (%)	CV (%) ^b^
LOQQC	1.01	0.95 ± 0.021	94.60	2.21
LQC	2.90	2.81 ± 0.088	96.90	3.13
MQC	1000.00	981.01 ± 9.33	98.10	0.95
HQC	2000.00	1979.01 ± 5.00	98.95	0.25

Values (Mean ± SD) are derived from 6 replicates:

^a^ Accuracy (%) = Mean value of [(mean observed concentration)/(theoretical concentration)] × 100

^b^ Precision (%): coefficient of variance (percentage) = standard deviation divided by mean concentration found × 100; Theoretical contents; LOQQC: 1.01 ng/ml, LQC: 2.90 ng/ml; MQC: 1000 ng/ml; and HQC: 2000 ng/ml

#### Ruggedness

A complete batch of DOX was analyzed (using different analysts, columns and solution) for precision in order to determine ruggedness of the method. The % accuracy (mean = 94.60–98.95) and % correlation of variance (mean = 0.25–3.13) for drugs (n = 6) is shown in Table 2b.

#### Matrix effect

It has been observed that the endogenous compounds (present as normal part of the sample) sometimes co-elute with actual drug and may disturb the peak retention position from its actual position. The matrix effect calculated for DOX (A/B × 100) at different levels showed a %CV (n = 6) of 3.12 for LQC and 3.37 for HQC whereas the %CV < 5 is an indication of lack of matrix effect. With the use of 5% formic acid (protein precipitating agent), DOX exhibited no ion suppression or enhancement as performed in post-column infusion method (using LLE).

#### LOD and LOQ

Spiked plasma samples (diluted with DOX standard till signal-to-noise ratio reached from 3 to 10) estimated for LOD and LOQ showed a value of 0.070 and 0.141 ng/ml; respectively.

#### Ex vivo stability

Two levels of QC (LQC and HQC) were studied for Ex vivo stability at different storage conditions such as long term, freeze–thaw, bench-top and post-processing stability as shown in Table 3. Stability results for % DOX recovery were observed as; long term stability (1 month) for LQC (98.59%) and HQC (98.13%), freeze–thaw stability (after 1, 2 and 3 freeze thaw cycles) for LQC (96.48–98.59%) and HQC (98.91–99.40%), bench top stability (24 h) for LQC (98.59%) and HQC (99.02%) and post processing stability of 99.60 and 99.14% for LQC and HQC.

**Table 3:** Ex vivo stability data for doxorubicin (DOX)

Conditions	LQC (2.90 ng/ml)	HQC (2000.00 ng/ml)
Long term stability; recovery (ng) after storage (− 40 °C)		
Previous day	2.83 ± 0.05	1989.04 ± 5.16
30th day	2.79 ± 0.04 (98.59%)	1951.78 ± 19.12 (98.13%)
Freeze–thaw stress; recovery (ng) after freeze–thaw cycles (− 40 to 25 °C)		
Pre-cycle	2.84 ± 0.03	1988.10 ± 4.19
First cycle	2.80 ± 0.04 (98.59%)	1976.19 ± 12.91 (99.40%)
Second cycle	2.78 ± 0.04 (97.88%)	1972.54 ± 13.16 (99.22%)
Third cycle	2.74 ± 0.05 (96.48%)	1966.44 ± 13.67 (98.91)
Heating–cooling stress; recovery (ng) after heating–cooling cycles (50–4 °C)		
Pre-cycle	2.85 ± 0.03	1989.09 ± 5.90
First cycle	2.80 ± 0.04 (98.25%)	1981.61 ± 11.95 (99.62%)
Second cycle	2.76 ± 0.03 (96.84%)	1974.01 ± 12.04 (99.24%)
Third cycle	2.72 ± 0.03 (95.44%)	1963.02 ± 13.02 (98.69%)
Bench top stability; recovery (ng) at room temperature (25 °C)		
0 h	2.84 ± 0.03	1988.49 ± 11.01
24 h	2.80 ± 0.05 (98.59%)	1969.10 ± 12.31 (99.02%)
Post processing stability; recovery (ng) after storage in auto sampler (4 °C)		
0 h	2.85 ± 0.03	1979.02 ± 7.91
24 h	2.81 ± 0.04 (99.60%)	1962.01 ± 14.02 (99.14%)

Values (Mean ± SD) are derived from six replicates. Figures in parenthesis represent analyte concentration (%) relative to time zero. Theoretical contents; LOQQC: 1.01 ng/ml, LQC: 2.9 ng/ml; MQC: 1000 ng/ml; and HQC: 2000 ng/ml

### Pharmacokinetic studies (PKs)

**Fig. 8:**
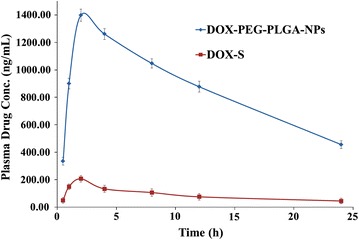
Plasma drug concentrations verses time profiles of DOX in male wistar rats after 10 mg/kg single dose of DOX-S (oral) and DOX-loaded-PEG-PLGA-NPs (oral)

Non-compartmental method (model independent) where plasma drug concentration vs. time profile for DOX-S as well as PEGylated-DOX-PLGA-NPs was depicted (single oral dose), as shown in Fig. 8. The C_max_ and T_max_ were found with the help of plasma drug concentration vs. time plot and the AUC (area under the curve) was determined through trapezoidal method.

For DOX-S the C_max_ attained was 206.2 ± 12.01 ng/ml whereas for PEGylated-DOX-PLGA-NPs it was 1398.19 ± 24.09 ng/ml at P < 0.001 and hence due to high C_max_ alongwith a lower elimination rate constant, a higher AUC_0−t_ was observed for PEGylated-DOX-PLGA-NPs (20577.90 ± 256.34 ng h/ml) compared to DOX-S (2130.75 ± 49.56 ng h/ml), as shown in Table 4.

**Table 4:** Pharmacokinetic parameters of DOX after single oral dose of DOX-S and PEGylated-DOX-PLGA-NPs (mean ± SD; n = 6)

Parameters	C_max_ (ng/ml)	T_max_ (h)	t_1/2_	AUC_0−t_ (ng h/ml)	AUC_0−∞_ (ng h/ml)	K_eli_ (h^−1^)	Rel Bio
DOX-S	206.2	2.00	17.08	2130.75	3232.49	0.04	
PEGylated-DOX-PLGA-NPs	1398.19***	2.00	36.64	20577.90***	44664.33***	0.02	9.7

*Rel Bio* relative bioavailability

** p < 0.01

*** p < 0.001

Higher C_max_ attained for PEGylated-DOX-PLGA-NPs is due to efficient NPs absorption, resulted due to small particle size and hydrophobic surfaces which enhance the interaction with enterocytes and Peyer’s patches and subsequently improved uptake and accessibility to systemic circulation [22, 54]. The prolonged C_max_ imparts a sustained in vivo release property to the drug (in agreement with in vitro release profile) whereas a higher AUC_0–∞_ as observed enhanced the oral bioavailability (~ 9.7 fold) for PEGylated-DOX-PLGA-NPs as compare to DOX-S, as shown in Table 4 (P < 0.001). As per literature reports, protection from hostile environment provided by encapsulation, enhanced permeation across intestine and direct uptake by enterocytes (bypass of first pass metabolism and P-gp transporters) results a higher absorption and bioavailability for PEGylated-DOX-PLGA-NPs [25, 28, 53].

Furthermore, numerous literatures have reported a rapid plasma drug clearance for doxorubicin solution i.e. i.v. hence a high as well as a frequent dosing is required at times which may lead to adverse effects [55–57]. In the study we developed PEGylated-DOX-PLGA-NPs whereby a plasma drug concentration for extended period of time was achieved. The study is in concordance with previous reports where an extended plasma drug concentration was observed for NPs such as; a comparable AUC for paclitaxel-loaded-NPs as compared to i.v. administered Taxol, enhanced AUC value for epirubicin-incorporated PEGylated-Nanoparticles compared to epirubicin i.v. solution etc. [27, 58].

Surface modification led to further improvement in PK profile of EPI. EPI-PNPs demonstrated significantly high oral bioavailability (AUC_0–∞_, significantly high P < 0.05) when compared with plain DOX-NPs, reported in previous work [53], EPI-MNPs also showed higher oral bioavailability however difference was extremely significant (P < 0.001). High oral bioavailability of DOX through PLGA-NPs than demonstrated by plain DOX-NPs can be supported with the mucus penetration ability of PEG, thus high diffusivity of nanoparticles in mucus gel layer of intestine hence reaching closer proximity to absorptive epithelial layer resulting in more absorption [20, 33]. The UPLC/ESI-Q-TOF–MS/MS bioanalytical method successfully developed and validated with linearity r^2^ ≥ 1.00–2500.00 ng/ml and applied to pharmacokinetic analysis in future. This method has advantages over other reported method that it contains very low concentration for detection of DOX in plasma (i.e. LLOQ 1.00 ng/ml). Literature for DOX-plasma-quantification is though available [42–46], but they suffer major drawbacks such as; no individual or separate method for DOX plasma determination, lack of sensitivity and ability to determination at nanogram level. UPLC-ESI-Q-TOF–MS/MS method has been developed and validated successfully with exhibit rapid, selective, sensitive and robust parameters for the quantification of DOX-PEG-PLGA-PNPs in rat plasma. The developed method reported no carry over effect and to quantify DOX in the plasma up to concentration (nanogram level) in developed NPs. The results of this study also shows; PEGylated-DOX-PLGA-NPs may be a potential alternate to improve DOX-oral bioavailability, achieving maximum therapeutic effect and to reduce the need for infusion equipment as well as hospitalization. However, to prove its clinical efficacy, extensive research studies in terms of safety and efficacy still needs to be conducted.

## Conclusion

PEGylated-DOX-PLGA-NPs were successfully developed and evaluated for their potential in oral delivery which exhibited significantly better in vitro and in vivo activities as well as a higher bioavailability compared to oral DOX-S. In addition, a rapid, potential, selective and sensitive UHPLC/ESI-Q-TOF–MS/MS was developed for DOX-plasma-quantification (detection limit up to picogram level), after oral administration in Wistar rats. The results; recovery of plasma analyte after extraction procedures (> 84.89%), linearity, accuracy and precision alongwith stability (bench-top, long term, freeze thaw stability and post processing stability) and matrix effect, were found in acceptable range. Finally, the developed method was successfully applied to quantify the amount of drug in plasma after in vivo administration in Wistar rats whereby an acceptable range for precision and accuracy was noted.
